# Impact Damage Localisation with Piezoelectric Sensors under Operational and Environmental Conditions

**DOI:** 10.3390/s17051178

**Published:** 2017-05-22

**Authors:** Mohammad Saleh Salmanpour, Zahra Sharif Khodaei, M. H. Ferri Aliabadi

**Affiliations:** Department of Aeronautics, Imperial College London, Kensington, London SW7 2AZ, UK; z.sharif-khodaei@imperial.ac.uk (Z.S.K.); m.h.aliabadi@imperial.ac.uk (M.H.F.A.)

**Keywords:** structural health monitoring, temperature compensation, Lamb wave, CFRP, delay and sum, piezoelectric, vibration

## Abstract

Guided-wave structural health monitoring (SHM) systems with piezoelectric sensors are investigated for localisation of barely visible impact damage in CFRP plates under vibration and different thermal conditions. A single baseline set is used in a delay-and-sum algorithm with temperature correction for damage localisation in a large temperature range. Damage localisation is also demonstrated under transient thermal conditions, with signals recorded while the temperature is rapidly decreased. Damage severity due to successive impact events is studied under constant temperature. Damage is also localised when the plate is subjected to random vibration.

## 1. Introduction

Permanently-attached transducer networks are an important part of structural health monitoring (SHM) systems. These systems can be used for damage detection in carbon fibre-reinforced polymer (CFRP) composite materials commonly used in aircraft structures. SHM covers a wide range of approaches; this work is focused on active ultrasonic guided wave methods. In sparse array guided wave SHM, a network of attached ultrasonic transducers, typically piezoelectric lead zirconate titanate (PZT), generate and sense diagnostics signals, which are influenced by the material and geometric properties of the host structure. Lamb wave propagation can be used in delay-and-sum baseline comparison methods to detect and localise damage [1,2].

**Figure 1:**
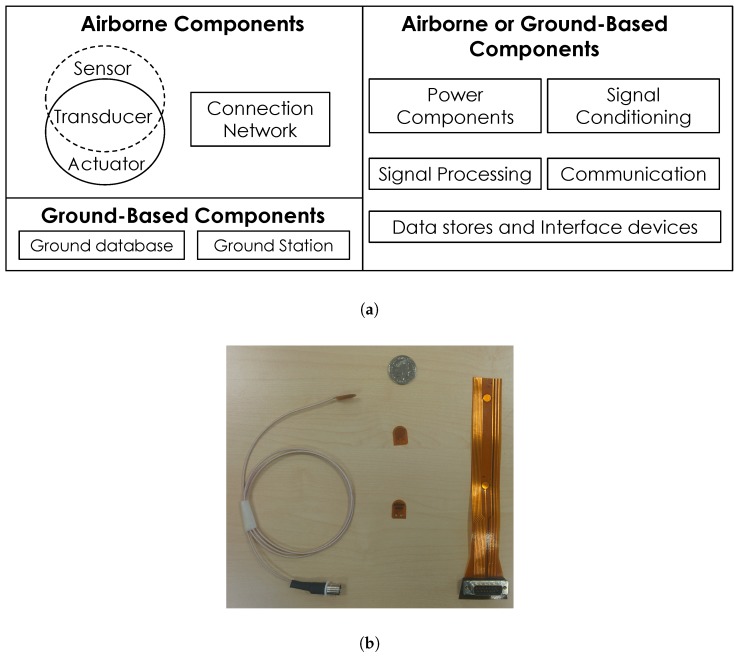
(**a**) SHM components (adapted from [25]); (**b**) piezoelectric transducers.

Typical airliners operate in a range of conditions; hence, airborne SHM components (see Figure 1) must be robust to relevant environmental conditions [3]. It has been shown that DuraAct (PI Ceramic, Lindenstraße, Germany) and SHM layer transducers are robust to thermal and vibration loading profiles required for airborne electronic components [4]. Additionally, SHM performance must also be demonstrated under a range of environments. Several promising systems have been demonstrated under laboratory conditions [1,2,5,6,7,8,9,10,11]. Researchers have investigated the effects of temperate [12,13,14,15,16], humidity [17] and vibration loading [18] on damage detection systems.

Konstantinidis et al. [19] investigated temperature effects for guided wave baseline comparison and found that the error is dominated by stiffness change in the host. Michaels [1] proposed an optimal baseline selection method (OBS). For an aluminium notch damage used in the study, she noted that a temperature match within 2 and 0.5 °C is required for detection and localisation respectively [1]. Hence, for damage localisation in the temperature range −50 to 60 °C, a minimum of 109 baseline sets is required. For a four-transducer pitch-catch network, this necessitates more than 1300 signal recordings. Croxford et al. [20] increased the temperature range by utilising a signal stretching approach, similar to that developed by Lu and Michaels [21]. Wilcox et al. [22] combined OBS with baseline signal stretching (BSS), reducing the number of required baselines. Wang et al. [23] developed an adaptive linear neuron temperature compensation method for Lamb wave damage detection, allowing a compensation temperature range of −40 to 80 °C with baselines at 12 °C intervals. More recently, Fendezi et al. [24] proposed a data-driven approach to reconstruct signal-correcting amplitude and phase in a BSS-based method using a library of correction factors. This was used to localise impact damage in a CFRP panel with a temperature difference of up to 22 °C. However, these researchers have not reported on the reliability of SHM systems under real operational conditions including vibration loads; in particular, the effects of operational conditions on the integrity of the integrated transducers.

The main novelty of this paper is damage localisation under operational environments; vibration, extreme steady state and transient temperature conditions, with only one baseline. Damage severity is also investigated with multiple impact events at a range of frequencies. First, the environmental conditions and experimental procedures are defined. Subsequently, temperature compensation results are presented for a CFRP plate showing localisation of barely visible impact damage (BVID) between −50 and 60 °C using the delay-and-sum method. Results are also presented for transient temperature conditions with signals recorded while the plate temperature was rapidly reduced. Localisation is also investigated under vibration loading on a CFRP plate. Finally, damage severity results on two different CFRP plates are presented.

## 2. Environmental Test Conditions

Environmental test conditions were chosen to replicate the possible on the ground conditions of a European regional aircraft.

### 2.1. Temperature

The MIL-STD 180G [26] gives low temperature in most of Europe as ‘Basic Cold C1’, with daily air temperature ranging between −32 and −21 °C, (−33 to −25 °C induced). Parts of northern Europe are classified as ‘Cold C2’ where induced temperatures range between −46 and −37 °C. It should be mentioned that the extreme low temperature in Europe is given as −55 °C in Russia occurring in a 15-year period [26]. The most extreme operating condition for an SHM systems was deemed to be within the Cold C2 classification with the low test temperature set at −50 °C.

High temperature in Europe is given as ‘Basic Hot A2’ to ‘Intermediate A3’, with an air temperature of 30 to 43 °C (30 to 63 °C induced) and 28 to 39 °C (28 to 58 °C induced), respectively [26]. The highest temperature ever recorded in Europe is given as 55 °C. The highest operating test temperature was set at 60 °C in these tests.

**Figure 2:**
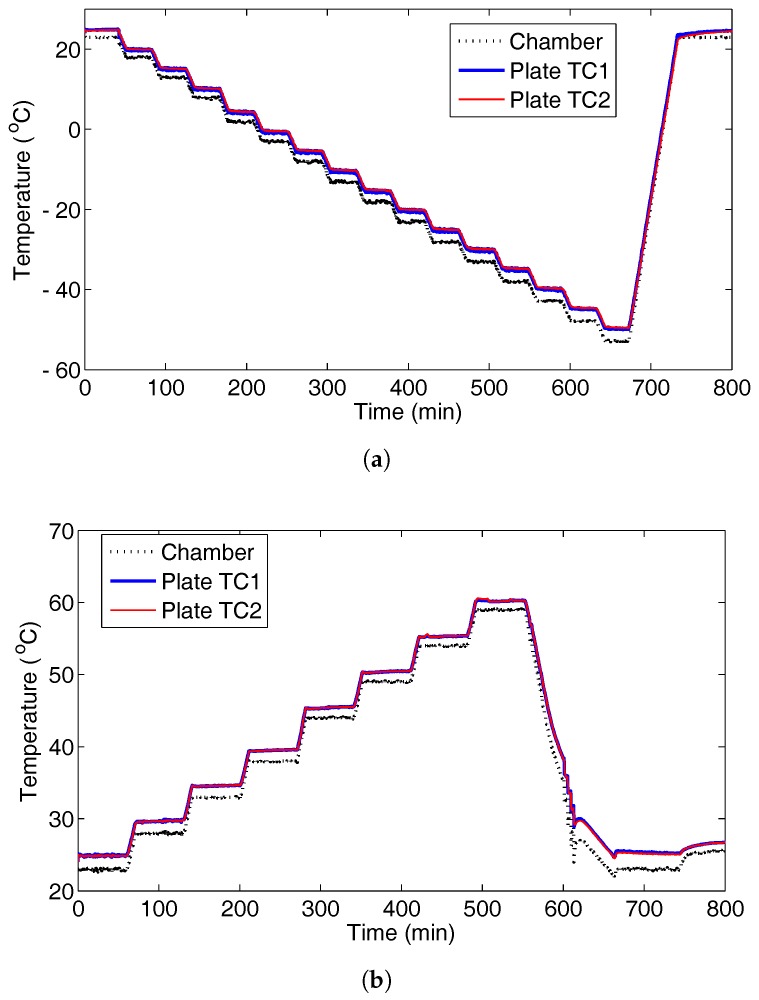
Recorded temperature profile for the chamber and two points on the plate (Thermocouple TC1 and TC2) for the steady state thermal tests: (**a**) low temperature; (**b**) high temperature.

For the steady state thermal tests, temperature was varied between the minimum and maximum values, in steps of 5 °C; see Figure 2. Once the structure temperature had stabilised, ultrasonic signals were recorded 10 times and averaged.

**Figure 3:**
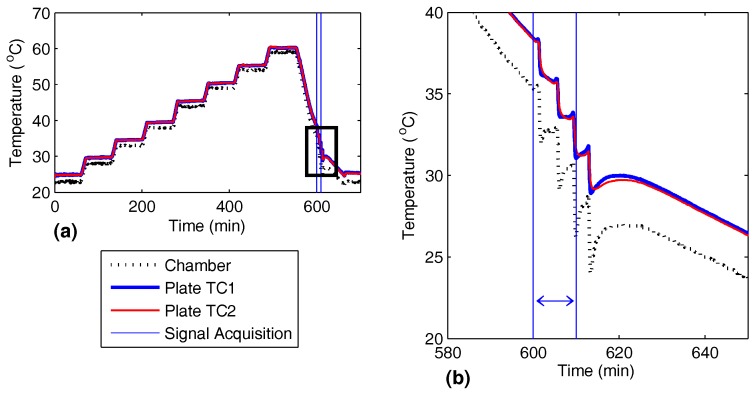
Rapidly decreasing temperature while the signal was recorded for the transient thermal tests. (**a**) Temperature profile for the chamber and two points on the plate (TC1 and TC2), (**b**) zoomed in temperature profile while signal was recorded.

In addition to signals recorded in a steady state, recordings were made with transient plate temperature. While the signal was being recorded, the test chamber temperature was reduced at 5 °C/min from 35 to 26 °C, as shown in Figure 3.

### 2.2. Vibration

**Figure 4:**
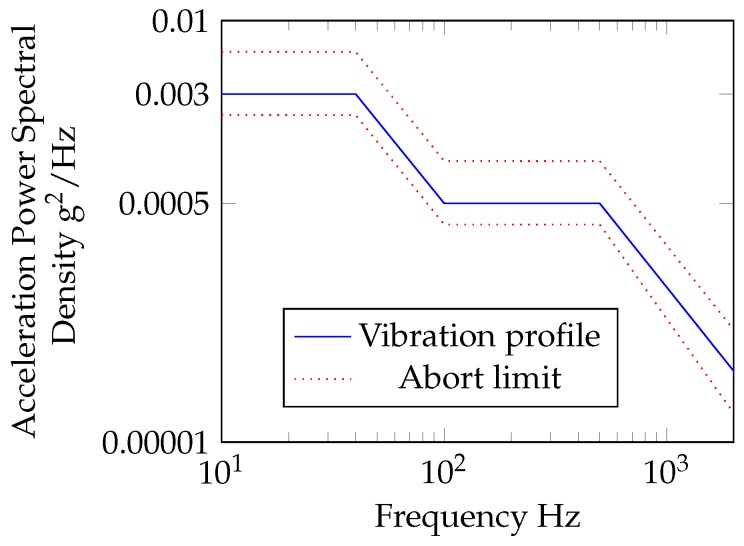
Random vibration profile, APSDprofile slopes −6 dB/OCT.

Depending on equipment location, RTCA DO-160 [3] specifies a certain vibration profile. For a turbojet (and turbofan), sinusoidal tests are required for engine locations; random vibration tests are required for all other locations. The standard random vibration test must be performed with the sample fixture representative of the actual operational structure. Ground-based SHM will be performed while the aircraft is stationary on the ground. For this, the in-flight “instruments and equipment rack” vibration profile was chosen. The vibration profile on the sample was controlled with a closed feedback loop and maintained in the limit shown in Figure 4.

## 3. Experimental Set-Up

Ultrasonics signals prior, during and after each test were generated and recorded, each transducer used in turn as the actuator and sensor (pitch catch). This was done using an NI-PXIe 5412 arbitrary voltage generator (National Instruments, Austin, TX, USA), an NI-PXIe 5105 digital oscilloscope (National Instruments, Austin, TX, USA) and a Pickering 40-726 switching card with a maximum output voltage amplitude of 12 volts (Pickering Interfaces, Clacton-on-Sea, UK). Ultrasonic Lamb waves were excited with a five cycle Hanning tone-burst with central frequency swept between 50 and 300 kHz in 50-kHz steps, and the signal was also recorded at 75 kHz. This ensured signals were available at both low (50 kHz) and high (300 kHz) frequencies with dominant $A_{0}$ and $S_{0}$ modes, respectively. The response was sampled at 60 MS/s for 0.001 s. Each recording was repeated 10 times, bandpass filtered and averaged.

### 3.1. Vibration Set-Up

**Figure 5:**
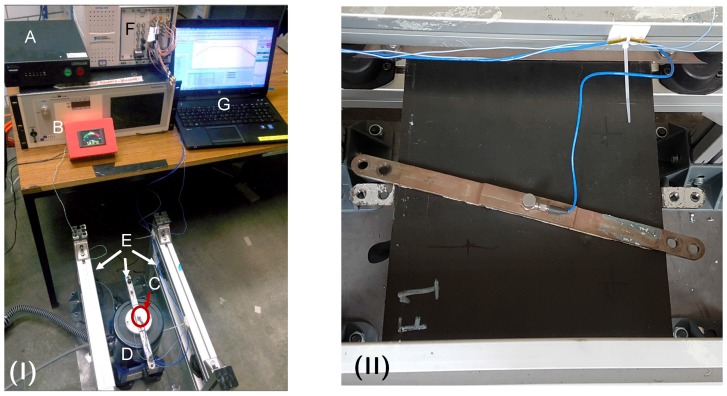
(**I**) Vibration set-up: (A) controller, (B) shaker power amplifier, (C) control accelerometer, (D) shaker, (E) fixture, (F) signal acquisition and generator system (G) workstation; (**II**) close up of the plate fixture.

The vibration set-up consisted of a TMS 2110E shaker (The Modal Shop, Sharonville, OH, USA) driven with a 2050E09-FS power amplifier (The Modal Shop, Sharonville, OH, USA). This was controlled with a Spider 81-B control and acquisition unit (Crystal Instruments, Santa Clara, CA, USA). A PCB Piezotronics 352C33 high sensitivity accelerometer (PCB Piezotronics, Depew, NY, USA) was used for the control measurements on the sample. Three fixture clamps were 15 cm apart, replicating stiffener bays, with the shaker coupling at the centre shown in Figure 5. Plate vibration was in the out of plane direction. A frequency response function was recorded for each sample, which was used in a feedback loop to achieve the required APSD profile.

The pristine baseline signal before impact was recorded under vibration, while the current signals under vibration were recorded after the plate was impacted. These were recorded at ambient lab temperature on separate dates.

### 3.2. Temperature

**Figure 6:**
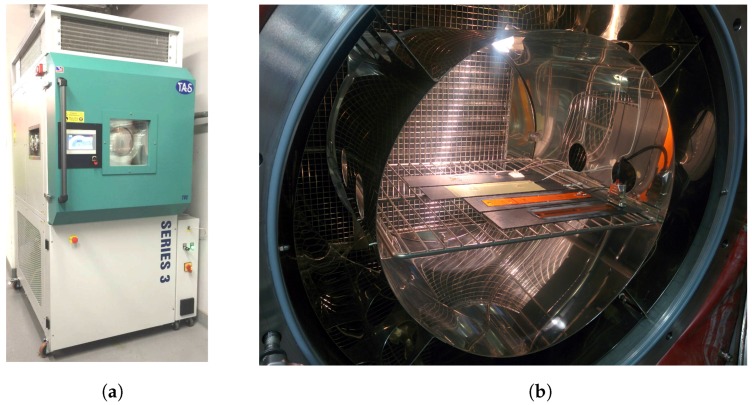
TAS Series 3 test chamber (**a**); test section (**b**).

The plate was exposed to environmental test profiles with a TAS Series 3 temperature, climatic and pressure chamber (Temperature Applied Sciences, Worthing, UK) shown in Figure 6. The chamber temperature was measured with a PRT (Platinum resistance thermometers) probe and with K-type thermocouples on the plate. Plate temperatures were recorded using an NI Compact DAQ 9172 (National Instruments, Austin, TX, USA) and with an NI 9211 thermocouple module (National Instruments, Austin, TX, USA). Temperature and humidity output readings of the chamber were continually recorded during. It was found that at extreme low temperatures, the chamber temperatures reading was 5 °C below the plate temperatures. This was due to the chamber PRT probe being positioned away from the test section; the actual plate temperature was used as the target for the temperature profiles.

Each plate was conditioned with exposure and dwell between maximum and minimum temperatures to relieve any residual stresses, this was repeated at least 10 times. Each sample was then maintained at 70 °C for three hours to remove any residual moisture.

### 3.3. Plate Definitions

**Figure 7:**
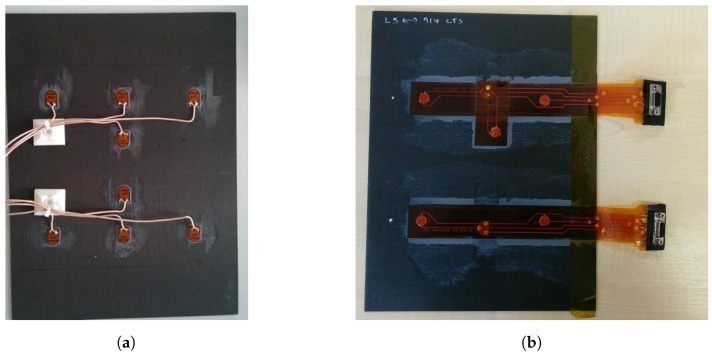
(**a**) DuraAct; (**b**) SHM layer plate.

DuraAct transducers and a manufactured SHM layer were permanently attached to separate CFRP host plates. The plates were manufactured using 16 unidirectional Hexply 914-TS-5-134 plies (Hexcel, Stamford, CT, USA) with stacking sequence [0,45,−45,90]${}_{2s}$ of 2 mm overall thickness. The transducers were bonded to the top surface with Hexcel Redux 312 film adhesive as shown in Figure 7.

**Figure 8:**
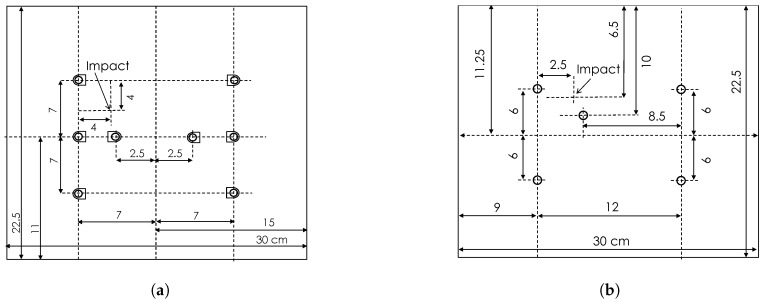
CFRP plate schematic with (**a**) DuraAct transducers and (**b**) SHM Layer, all distances in cm.

DuraAct transducer plate: Eight PIC DuraAct transducers were mounted centrally on a CFRP plate of a size 225 mm × 300 mm; see Figure 7a and Figure 8a. Each of these had PZT discs 10 mm × 0.2 mm (diameter × thickness) potted in proprietary resin covered with polyimide (Kapton) film, giving an overall dimension of 17 mm × 13 mm. These were soldered with RG178 cable (MIL-C-17G) (purchased from RS components Ltd., Corby, UK).

Smart layer plate: The SHM Layer was manufactured in-house as a composite layup. This consisted of double-sided flex circuit and PZT discs sandwiched in Kapton film with B-staged modified acrylic adhesive. The discs of 10 mm × 0.25 mm were covered with Kapton film on both sides and then mounted on a CFRP plate of a size 225 mm × 300 mm, shown in Figure 7b and Figure 8b. A D-sub connector was used with the RG178 cable (purchased from RS components Ltd., Corby, UK).

### 3.4. Impact Damage

The plates were impacted using a drop tower with a 10-mm radius hemispherical impactor head. The presence of damage was verified with a DolphiCam hand-held scanner; figures are presented in the damage severity Section 5.2.

The SHM layer plate was impacted multiple times with progressively increasing impact energy. Signals under environmental loading were recorded between the first and second impact events. The DuraAct plate was impacted multiple times, and signals recorded after each impact were used in investigating damage severity. The impact events are summarised in Table 1 and Table 2.

## 4. Damage Detection

This section provides a concise description of the detection method used and the strategy adopted to overcome environmental effects.

### 4.1. Localisation Method

**Figure 9:**
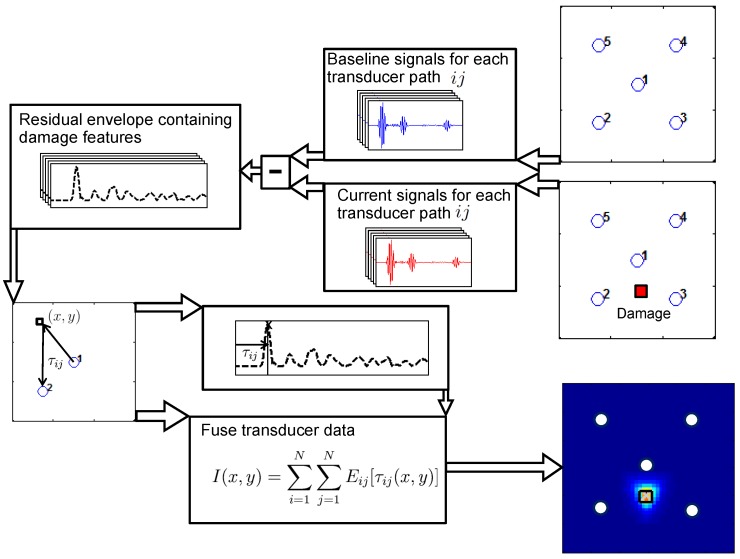
Main steps in the delay-and-sum method for damage localisation [27].

Guided wave propagation in real structures is very complex, and wave packets corresponding to geometric features are not readily identifiable. By subtracting signals recorded in the pristine baseline state $B_{ij}\left[t\right]$ from the current signals $C_{ij}\left[t\right]$, it is possible to isolate the damage scatter waves. The envelopes of these residuals $R_{ij}\left[t\right]$ are referred to as the residual envelopes $E_{ij}\left[t\right]$, for each sensor-actuator path ij:

(1) $$\begin{array}{cc} E_{ij}\left[t\right] & =env(R_{ij}\left[t\right])\\ & =|R_{ij}\left[t\right]+iH\left(R_{ij}\left[t\right]\right)| \end{array}$$

where H is the Hilbert transform. The delay-and-sum algorithm [1,2] then uses the time of arrival $\tau_{ij}\left(x,y\right)$ at each point (x,y) to build a 2D damage index map I(x,y) from the residual envelopes, highlighting the position of damage:

(2) $$\begin{array}{c} I\left(x,y\right)=\sum_{i=1}^{N}\sum_{j=1}^{N}E_{ij}\left[\tau_{ij}\left(x,y\right)\right] \end{array}$$

where *N* is the number of transducers. It should be noted that damage can only be localised accurately within the transducer area [27]. An outline of the detection method used is shown in Figure 9.

### 4.2. Temperature Correction

A difference in the operational temperatures when recording diagnostic signals can bring about a non-damage-related residual [19,28]. This can significantly reduce the detection and localisation accuracy [1]. In this paper, stretch-based temperature correction is applied to correct the velocity changes due to temperature [13]:

(3) $$\begin{array}{c} R_{ij}^{k}\left[t\right]=B_{ij}\left[t\right]-C_{ij}\left[t-\zeta_{k}t\right]. \end{array}$$

However, instead of assuming a single stretch factor for the entire signal, a range of stretch factors $\zeta_{k}$ is iteratively applied, reducing the residual level at each point in the signal:

(4) $$\begin{array}{c} E_{ij}^{k}\left[t\right]=min\left(E_{ij}^{k-1}\left[t\right],env\left(R_{ij}^{k}\left[t\right]\right)\right) \end{array}$$

At each step *k*, the residual level is necessarily lower than in the previous step. The obtained temperature residual can then be used as the input for the delay-and-sum algorithm. It must be noted that knowledge of current temperature is not required.

### 4.3. Vibration Filtering

**Figure 10:**
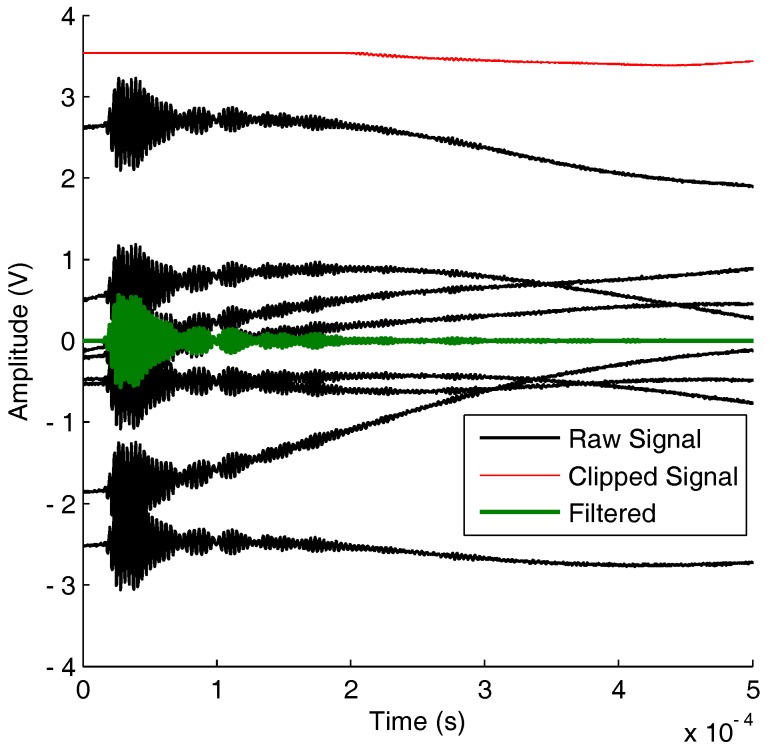
Signal recorded under random vibration highlighting signal clipping. Actuation at 300 kHz on a DuraAct CFRP plate with eight transducers.

The main challenge in damage detection under random vibration was efficient removal of low frequency noise. A zero-phase Butterworth filter with a high-pass cut off above 2 kHz was used to remove vibration noise. However, it was found that the random low frequency noise had a higher amplitude than the diagnostic signals, as shown in Figure 10. This meant that the signal was clipped or even saturated if a recording voltage range close to the anticipated diagnostic signal amplitude was used. At times, this appeared similar to a DC offset; however, this could not simply be removed with AC coupling, as it had a finite period.

A hardware high-pass filter was a possible solution for the high amplitude low frequency noise. However, to utilise the same system used for the temperature tests, a larger vertical range was used at the expense of voltage resolution. Furthermore, it was found that outliers still existed outside this voltage range; hence, these were excluded from the signal averaging.

## 5. Results and Discussion

### 5.1. BVID Localisation

In this section, the results for BVID localisation on the SHM layer plate are presented. The damage localisation figures are normalised with the maximum damage index. Damage severity was also investigated for the DuraAct transducer and SHM layer plate.

#### 5.1.1. Steady State Thermal

**Figure 11:**
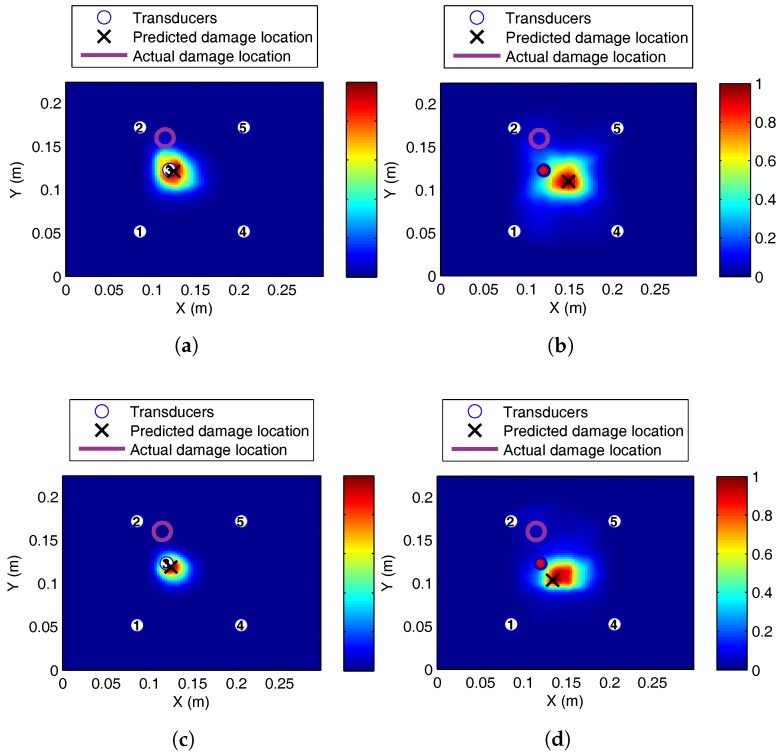
Barely visible impact damage (BVID) impact detection on the SHM layer CFRP plate at 50 kHz, baseline recorded at 25 °C: (**a**) 0 °C, five transducers; (**b**) 0 °C, four transducers; (**c**) 35 °C, five transducers; (**d**) 35 °C, four transducers.

There was significant reduction in the localisation accuracy when the current signals were recorded at increased or reduced temperature levels compared to the baseline level. Without temperature correction, the location of BVID was erroneously predicted to be near the centre of the plate for the symmetric four-transducer arrangement, as shown in Figure 11. The erroneous localisation shifted towards the location of the third transducers for the five-transducer case. This was the case for both increased and reduced temperatures.

**Figure 12:**
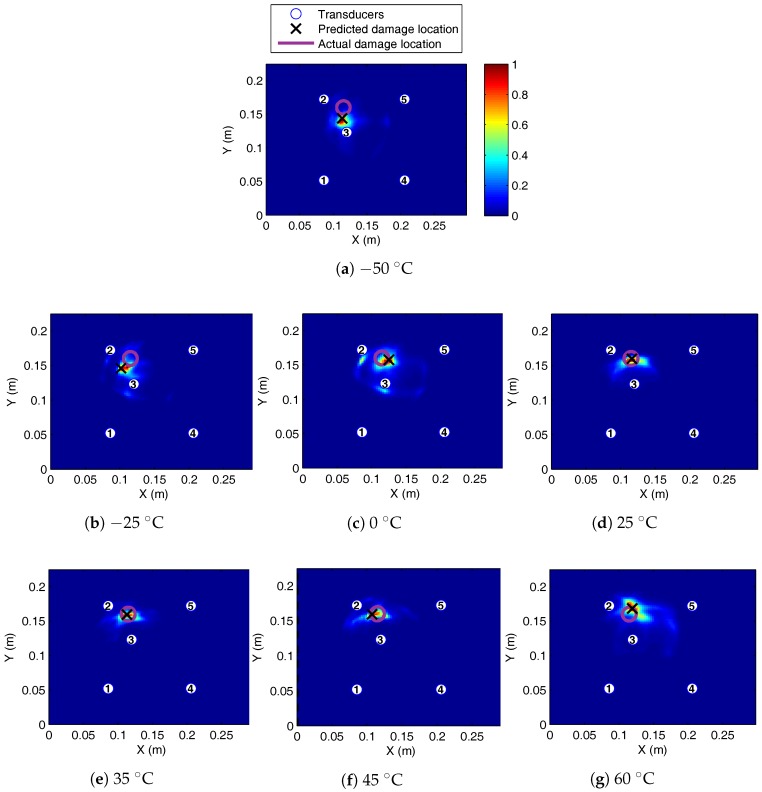
BVID localisation on the SHM layer CFRP plate at 50 kHz, baseline at 25 °C, with compensated current signal temperature recorded between −50 °C and 60 °C.

**Figure 13:**
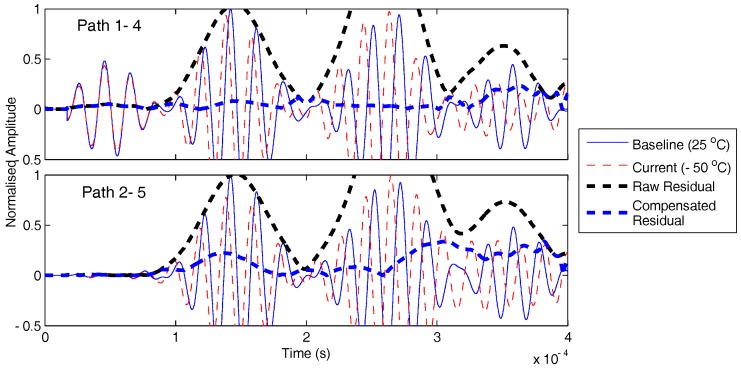
Current and damaged signal (50 kHz) for impacted SHM layer CFRP plate. Impact damage location far from the path 1–4 compared to the path 2–5.

With temperature correction, it was possible to localise damage both for 50- and 300-kHz excitation; see Figure 12. Considering two similar paths at 50 kHz, one far from damage (1–4) and another relatively closer to damage (2–5), it is clear that in both cases, the residual is significantly reduced from uncompensated (raw) case as shown in Figure 13. For the path closest to damage (2–5), there is higher residual at the first arrival, while for the path far from damage (1–4), the first arrival residual is small. The compensation scheme allows damage paths to be identified, extracting the damage scatter in the presence of significant non-damage residual.

Localisation accuracy was compared at different temperatures with temperature compensation for the four- and five-transducer network at 50-kHz excitation and is shown in Table 3. At this frequency of excitation, temperature compensations were effective between the full temperature range tested (−50 to 60 °C). As expected, accuracy reduced with increasing temperature different between the current and baseline temperature. However, four transducers under ambient conditions gave the largest localisation error. It should be noted that compensation did not cause this inaccuracy, as without correction, the absolute error was still 22.2 mm. The five-transducer network was consistently more accurate than the four transducers for 50-kHz actuation.

Temperature compensation was also effective for 300-kHz excitation, allowing localisation to within 2.5 cm between −5 and 35 °C, with the exception of 15 °C; see Table 4. It should be noted that only the four-transducer network had good performance at 300 kHz. The reduced temperature range can be associated with the reduced sensitivity of 300-kHz excitation to BVID. The relative amplitude of the residual was at least 50% lower compared to the A0 dominant excitation at 50 kHz. This is discussed in more detail in the damage severity section.

#### 5.1.2. Transient Thermal

In the previous section, signals were obtained at a range of temperatures in a steady state. That is, while signals were being recorded, temperature was held constant. In this section, temperature was varied while the signals were recorded.

**Figure 14:**
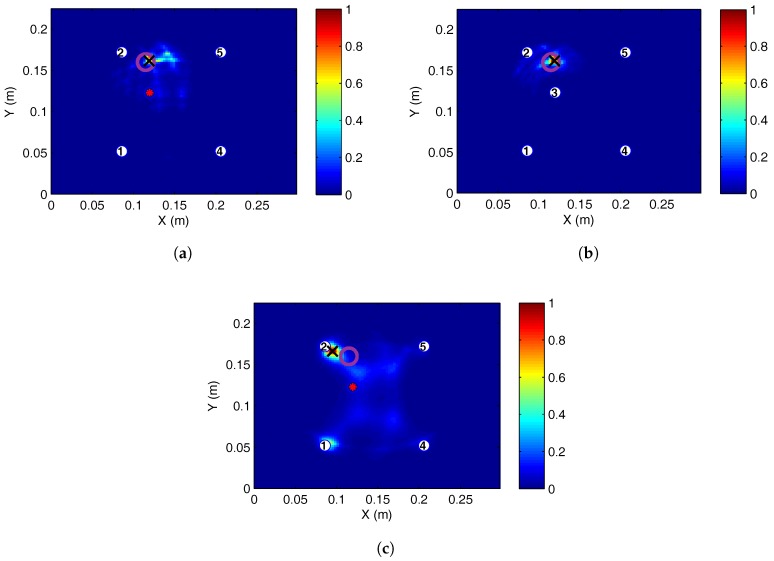
BVID impact localisation on the SHM layer CFRP plate, baseline at 25 °C with compensated current signal. Temperature was varied between 31 and 38 °C when the signal was being acquired: (**a**) 50 kHz, four transducers; (**b**) 50 kHz, five transducers; (**c**) 300 kHz, four transducers.

It was found that under transient thermal conditions, applying temperature compensation made it possible to localise BVID. This was because the compensation method did not assume a constant temperature, and a range of stretch factors was used. As before, the 50-kHz signal produced more accurate results, with the five-transducer arrangement having the least noise, as shown in Figure 14b. At 300 kHz, the localisation was closely matching that of current signal recordings at 35 and 30 °C steady state temperature.

In addition to the current temperature varying from the baseline, the effect of transient temperature is two-fold. Firstly, the temperature at which the signal for each path is recorded will vary within each dataset. Secondly, the plate temperature distribution will not be isothermal, adding to the complexity of wave propagation. The temperature compensation scheme was able to mitigate these effects.

#### 5.1.3. Vibration

**Figure 15:**
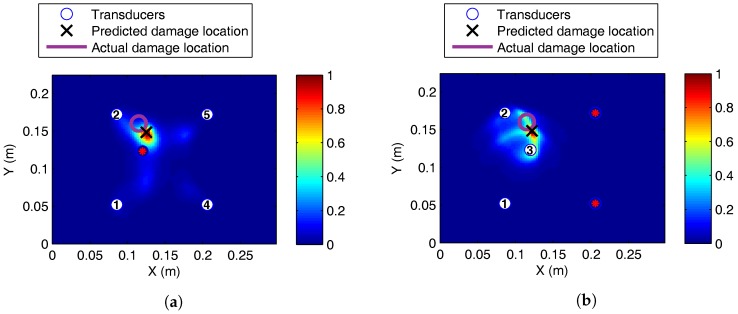
Damage localisation for BVID on the CFRP panel at (**a**) 300 kHz and (**b**) 50 kHz. Damage current and baseline signal recorded under vibration. For (**b**), 50 kHz only using transducer paths that do not cross a fixture clamp. (**a**) Three hundred kilohertz, four transducers; (**b**) 50 kHz, three transducers.

Damage was localised accurately with 300-kHz excitation during the vibration, using four transducers as shown in Figure 15a. At 50 kHz, localisation was only possible using transducers that did not cross the vibration fixture clamp; see Figure 15b.

It may appear that random vibration loading was the main reason for poor performance at 50 kHz. However, boundary conditions imposed by the vibration fixture had a significant impact on the wave propagation. It should be noted that to impact the plate in the drop tower, it had to be removed from the vibration fixture. The plate was then replaced in the fixture, with no way of ensuring identical boundary conditions. A real structure will be fixed in place, and there will not be non-damage-related changes in the boundary conditions within the interrogation area. Boundary condition changes will often be associated with damage formation, e.g., stiffener de-bonding.

### 5.2. Damage Severity

**Figure 16:**
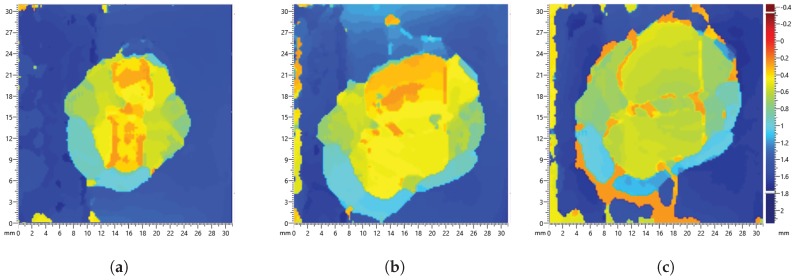
C-scan of BVID on the SHM layer CFRP panel for each impact event, with increasing energy (**a**) 5.68 J, (**b**) 6.41 J and (**c**) 7.84 J.

**Figure 17:**
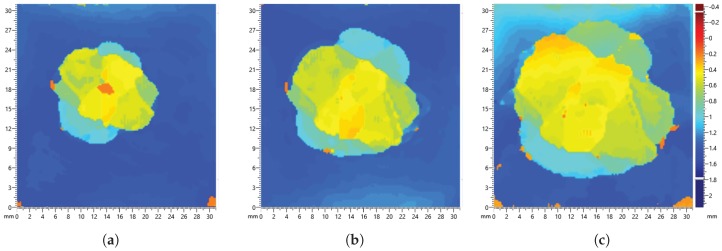
C-scan of BVID on the DuraAct CFRP panel for each impact event, with increasing energy energy (**a**) 4.8 J, (**b**) 6.41 J and (**c**) 7.84 J.

**Figure 18:**
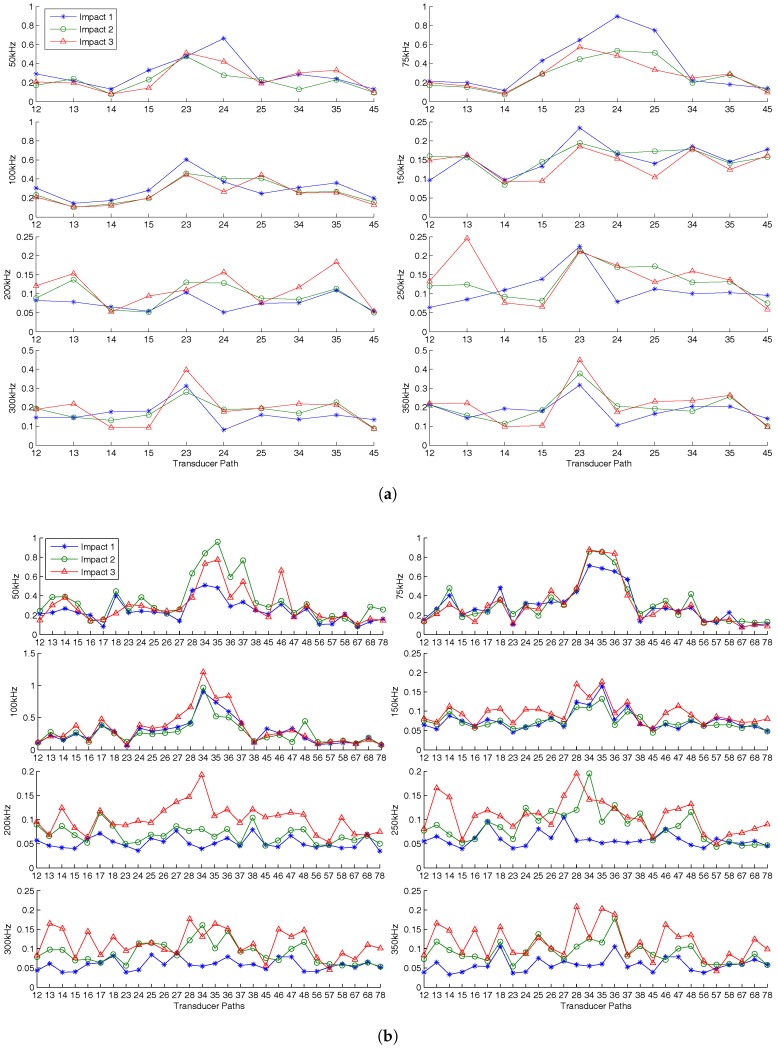
Normalised residual for the CFRP plate with (**a**) the SHM layer and (**b**) the DuraAct transducer after three impact events. Residual as a ratio of the largest amplitude in the waveform signal.

Damage severity was investigated with multiple impact events on the plate with increasing energy shown in Table 3 and Table 4. It can be observed from the C-scan images in Figure 16 and Figure 17 that there is an increase in damage size after each impact event. The first impact energy for the DuraAct plate was lower; hence, there is a larger range in damage size. The final impact on both plates caused visible damage. Residual signals containing damage scatter was obtained using baseline signals recorded before the first impact; the maximum values for each path are presented in Figure 18.

An important point to note is the sensitivity to damage, specifically comparing 300 kHz and 50 kHz. There exists dominant fundamental symmetric (S0) and antisymmetric (A0) modes at 300 kHz and 50 kHz, while excitation between these frequencies for both fundamental modes is produced without one being dominant. At 50 kHz, it can be observed that the maximum residual is more than double that of 300 kHz (0.66 and 0.32 respectively for the SHM layer); see Figure 18.

For the first impact on the DuraAct plate, the damage residual is very small (indistinguishable from noise) at 300 kH, while it is clearly distinguishable at 50 kHz. The first impact on the SHM layer plate was of higher energy, explaining the discrepancy between the residual levels in the two plates following the first impact as shown in Figure 18.

Overall, the least ambiguous indication for damage severity was at 200-kHz actuation. There was a consistent rise between the maximum residual levels between the impact events, as expected. However, this trend was more pronounced for the DuraAct transducer plate than the SHM layer. This can be put down to there being 80% more unique transducer paths available for comparison. The first impact was identifiable with lower residual on the DuraAct plate at frequencies above 200 kHz, as there was lower sensitivity at high frequencies to the least sever damage induced by the first impact.

As a consequence of increasing damage size and severity, it may be intuitive to assume damage scatter amplitude and hence the residual would increase with subsequent impact events. However, it was found that this was not the case for all transducer paths and frequencies. For frequencies other than 200 kHz, there was not a clear increase in the maximum residual due to subsequent impacts across all paths; see Figure 18. In the case of 75- and 100-kHz actuation, there was a reduction in residual for all but two paths with subsequent impacts.

This unpredictable trend in the scatter amplitude (hence residual amplitude) after successive impact events was also noted by [29]. This was attributed to impact events opening and closing the delamination in the laminate. A more compelling explanation could be the resonate energy blocking phenomenon [30].

It is suggested here that small severity/size BVID may produce larger amplitude scatter than an enlarged damage, due to its resonant frequency coinciding with the excitation. Lower severity damage is able to trap enough energy, in the form of standing waves when excited near resonance, allowing it to form a residual signal with amplitudes that match or exceed that of larger damage. That is, subsequent impact events shift the damage resonant frequency beyond the excitation frequency; hence less energy is trapped by the damage. This would be consistent with the sharp changes in resonant frequency and wave transmission coefficient due to damage size and depth predicted by [30].

It can be concluded that to characterise damage using scatter amplitude, excitation frequency far from the resonant frequencies of the expected damage type should be utilised. For the BVIDs in this work, this was at 200 kHz, as demonstrated by an increase in the residual amplitudes with successive impacts.

## 6. Conclusions and Future Work

Barely visible impact damage was localised on a CFRP plate with a single pristine baseline recorded at 25 °C in a temperatures range of −50 °C to 60 °C by applying temperature compensation. It was found that the A0 mode, dominant at 50 kHz, was most sensitive to the presence of BVID and more effective at localising damage. Damage localisation was also possible under transient thermal conditions with the plate temperature reducing at a rate of 5 °C/min while the signal was being recorded, as the temperature compensation did not assume constant temperature.

Under vibration loading, localisation of the damage was also possible at 50 kHz only if the change in boundary conditions due to the fixtures was avoided. Additionally, 300-kHz excitation was less sensitive to the fixture inconsistency

Damage severity and size could not reliably be determined from damage scatter amplitude. There was no clear trend in amplitude levels after successive impact events; this was associated with resonant wave trapping. It is suggested that to characterise damage severity with scatter amplitude, non-resonant excitation frequencies should be used. Frequency-dependant sensitivity levels to damage, damage severity and changing boundary conditions suggest that for localisation and characterisation of BVID, multiple frequencies must be utilised.

Future work will focus on determining more reliable metrics for damage characterisation, such as time of arrival or combining scatter amplitudes at different frequencies. This could also involve using hybrid approaches that combine electro-mechanical impedance data for characterising damage.

## Figures and Tables

**Table 1:** Multiple impact events on the SHM layer plate.

Impact Event	Energy (J)
1	5.68
2	6.41
3	7.84

**Table 2:** Multiple impact events on the DuraAct plate.

Impact Event	Energy (J)
1	4.8
2	6.41
3	7.84

**Table 3:** BVID localisation error on SHM layer CFRP plate (50 kHz), actual location (115,160) mm. Baseline recorded at 25 °C with the temperature-compensated current signal, using 5 or 4 transducers. * Indicates 4-transducer arrangement.

Temperature (°C)	Localisation (mm)		Absolute Error (mm)
	*x*	*y*	
−50	113.2	143.7	16.39
−25	101.3	145.9	19.7
0	125.2	161.6	10.3
25	116.2	159.4	1.3
35	113.2	159.4	1.9
45	107.3	159.4	7.7
60	116.2	170.6	10.7
−50 *	113.2	139.2	20.9
−25 *	101.3	148.2	18.1
0 *	125.2	157.2	10.6
25 *	137.1	154.9	22.7
35 *	122.2	163.9	8.2
45 *	119.2	163.9	5.7
60 *	119.2	168.4	9.4

**Table 4:** BVID localisation error on the SHM layer CFRP plate (300 kHz), actual location (115,160) mm. Baseline recorded at 25 °C with temperature compensated current signal, using 4 transducers.

Temperature (°C)	Localisation (mm)		Absolute Error (mm)
	*x*	*y*	
20	137.1	123.5	42.7
−15	131.1	123.5	39.9
−10	131.1	125.7	37.9
−5	116.2	141.4	18.6
0	116.2	141.4	18.6
5	110.3	143.7	17.0
10	116.2	139.2	20.8
15	160.9	143.7	48.7
20	116.2	145.9	14.2
25	101.3	159.4	13.7
30	95.4	163.9	20.0
35	95.4	166.1	20.6
40	86.5	51.6	112.1
